# Venous Thrombus Calcification over Time

**DOI:** 10.31662/jmaj.2025-0033

**Published:** 2025-03-28

**Authors:** Yusuke Namba, Koji Tokioka, Yusuke Kawai

**Affiliations:** 1 Department of Cardiovascular Medicine, Okayama City Hospital, Okayama, Japan

**Keywords:** thrombus, calcification, inferior vena cava, left common iliac vein, magnetic resonance imaging

A man in his 60s with obstructive pyelonephritis due to upper urinary tract urolithiasis diagnosed 7 years earlier and no thrombophilia risk presented with left lower-extremity edema. Contrast-enhanced computed tomography (CeCT) revealed a calcified thrombus extending from the inferior vena cava to the left common iliac vein (LCIV; Figure 1). A CeCT performed 4.5 years prior showed a non-calcified thrombus in the LCIV (Figure 2), and no anticoagulants were prescribed. Magnetic resonance imaging (MRI) performed 18 months prior showed a low-intensity area in an intermediate-intensity area. MRI performed on the day following the visit demonstrated that the low-intensity area had increased over time (Figure 3). His edema resolved following rivaroxaban administration, which was continued thereafter.

**Figure 1.**
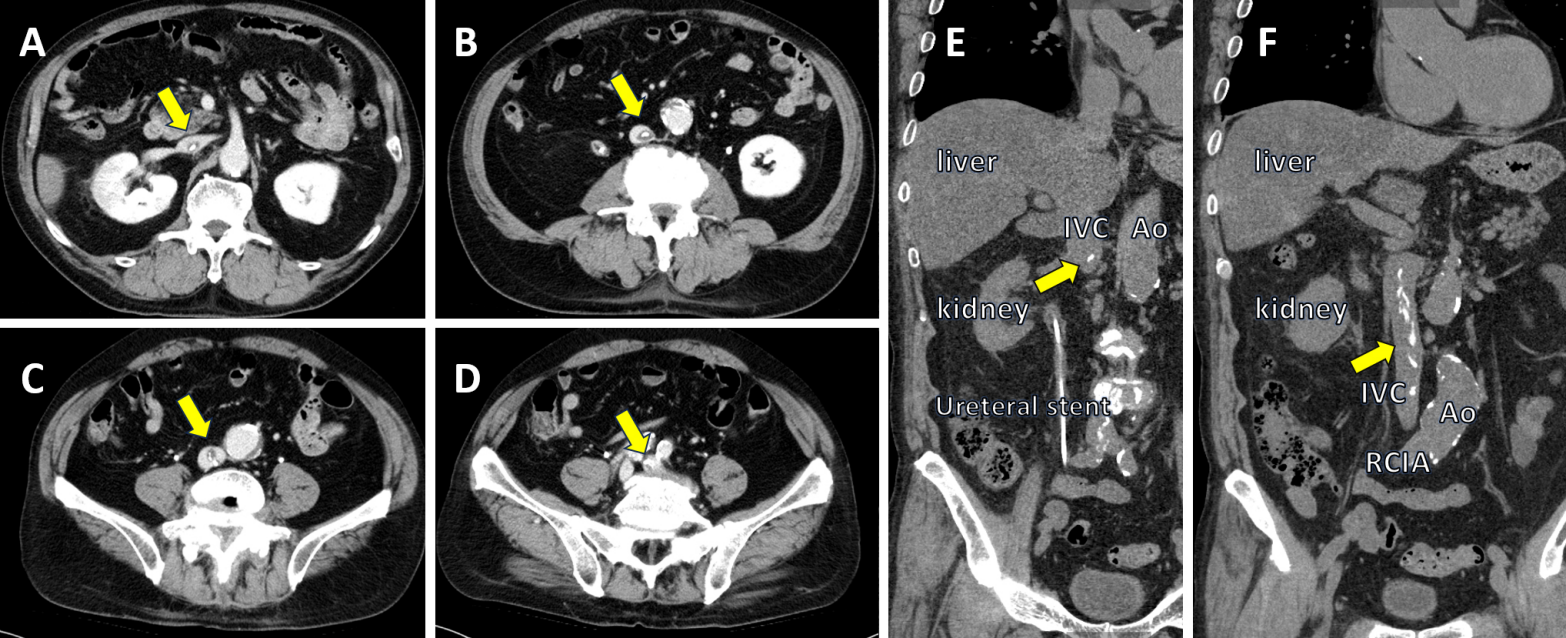
Contrast-enhanced computed tomography showing a calcified thrombus (yellow arrow) extending from the inferior vena cava to the left common iliac vein in axial images (A - D) and coronal images (E, F).

**Figure 2.**
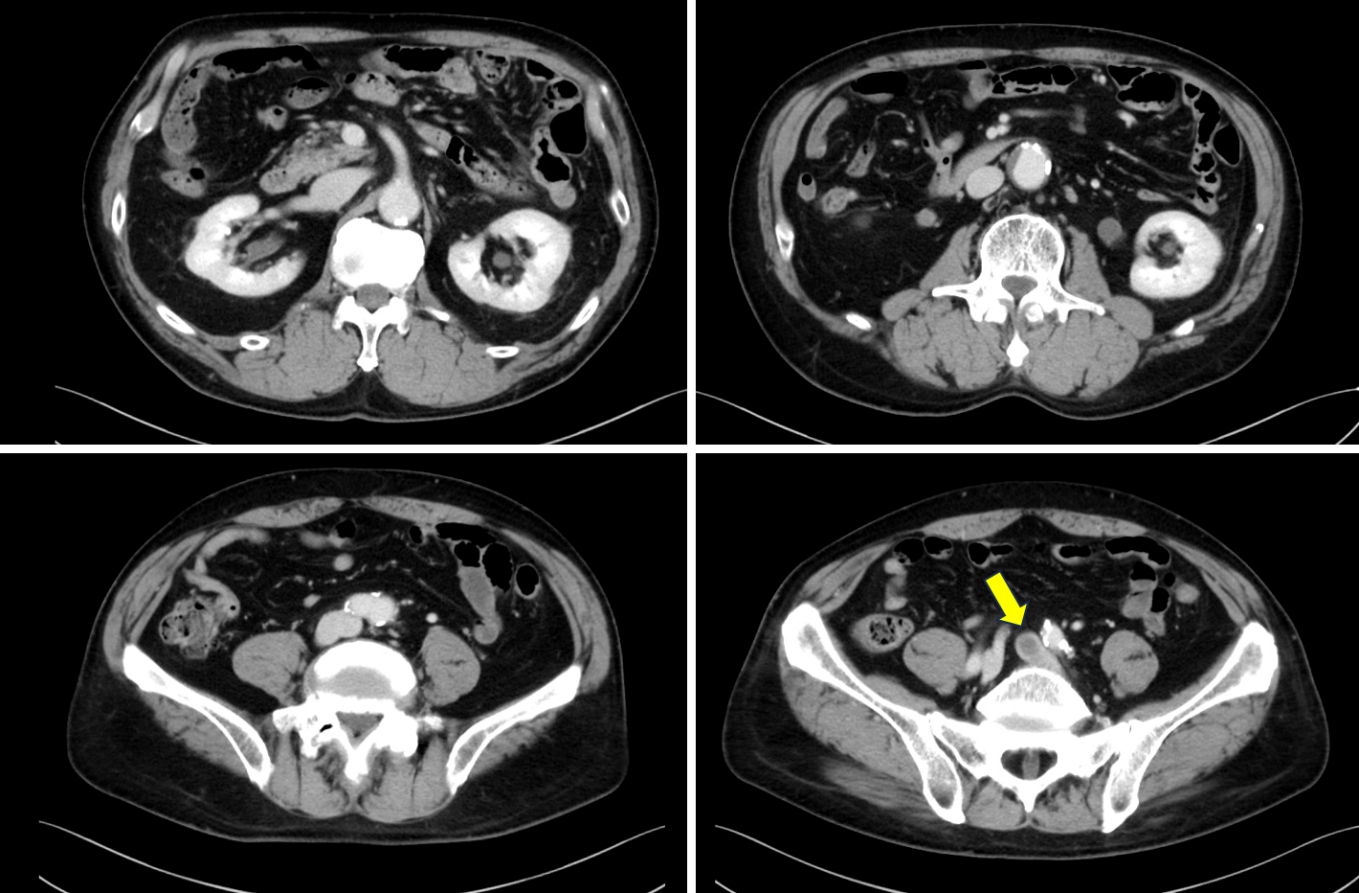
Past contrast-enhanced computed tomography showing a non-calcified thrombus in the left common iliac vein.

**Figure 3.**
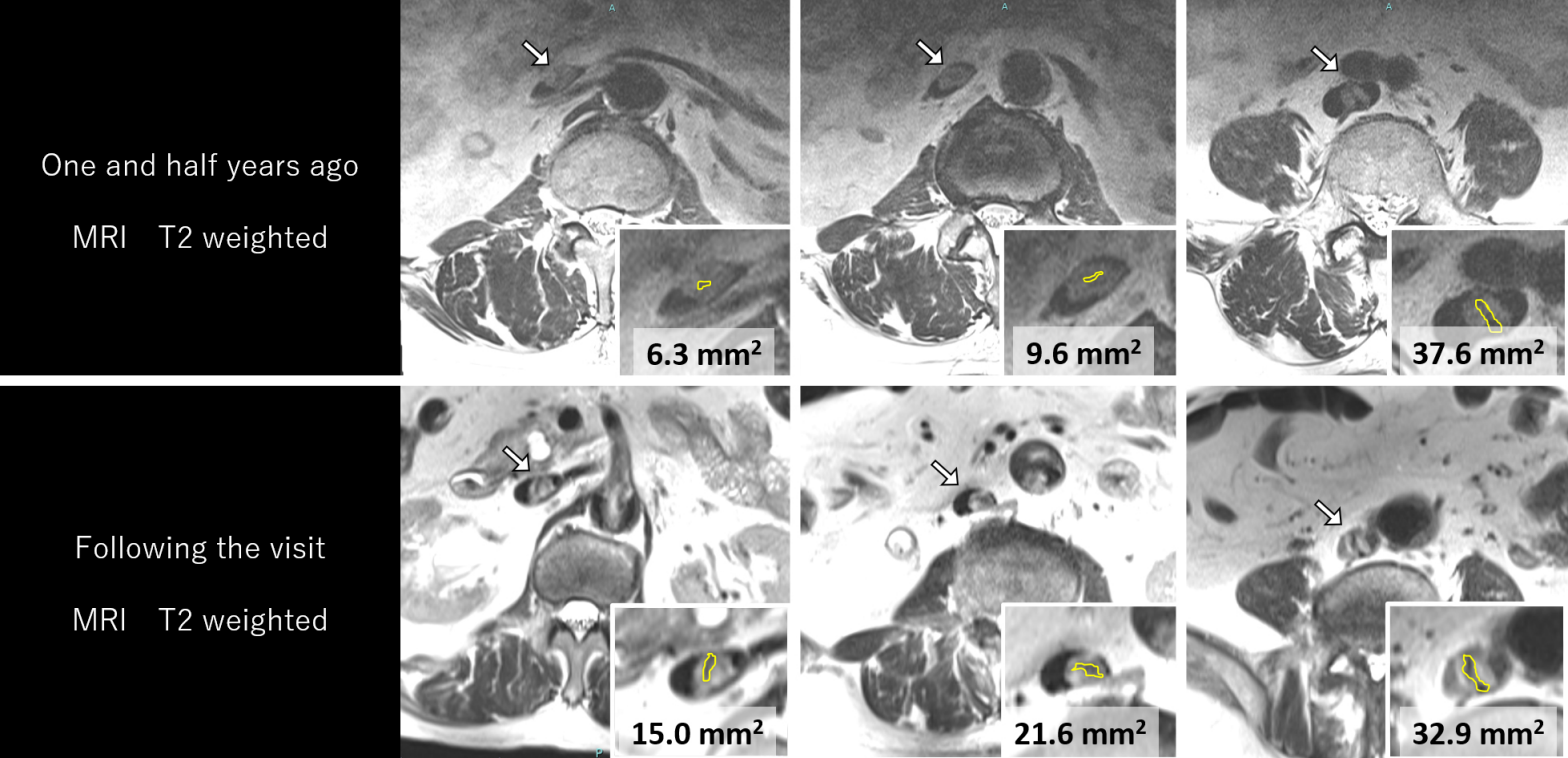
Increasing the low-intensity area (yellow enclosure and area value) over time by comparing magnetic resonance imaging performed 18 months ago and the day following the visit.

A previous report demonstrated inflammatory infiltrate and fibrotic evolution in pulmonary artery thrombi over time ^(1)^.

However, no study has assessed non-calcified thrombi that calcified over time. We believe this case is significant because it shows that an organized thrombus can calcify. Clinicians should treat deep vein thrombosis formation with anticoagulants to prevent thrombi formation and calcification.

## Article Information

### Conflicts of Interest

None

### Author Contributions

Yusuke Namba and Koji Tokioka managed the patient. Yusuke Namba wrote the original manuscript. Koji Tokioka and Yusuke Kawai reviewed and supervised the manuscript.

### Approval by Institutional Review Board (IRB)

IRB approval was not required for this report.

### Informed Consent

Consent to publish the details of the present case was obtained from the patient.
